# Composition equivalents of stainless steels understood via gamma stabilizing efficiency

**DOI:** 10.1038/s41598-021-84917-z

**Published:** 2021-03-08

**Authors:** Shuqi Zhang, Qing Wang, Rui Yang, Chuang Dong

**Affiliations:** 1 Key Laboratory of Materials Modification by Laser, Ion and Electron Beams (Ministry of Education), School of Materials Science and Engineering, Dalian University of Technology, Dalian, 116024 China; 2 School of Creativity and Art, ShanghaiTech University, Shanghai, 201210 China; 3 Institute of Metal Research, Chinese Academy of Sciences, Shenyang, 110016 China; 4 School of Materials Science and Engineering, Dalian Jiaotong University, Dalian, 116028 China

**Keywords:** Structural materials, Theory and computation

## Abstract

The phase-type of a stainless steel is generally predicted by equivalent equations in terms of a major austenitic (γ) or ferritic (α) stabilizer Ni or Cr. The present paper attempts to understand the equivalent methods in stainless steels via the slopes of the phase boundary lines separating γ and γ + α phase zones. The prevailing equivalent coefficients are well interpreted using the slope ratios of the alloying elements divided by that of Ni or Cr, after analyzing over one hundred common stainless steels. Different from traditional composition equivalents which evaluate γ stabilizers and α stabilizers separately; the new equivalent scheme provides a unified phase stabilizing parameter for all alloying elements in stainless steels. This parameter is defined as γ stabilizing efficiency. Its negative or positive sign indicates γ stabilizer or α stabilizer, and its value represents the stabilizing efficiency.

## Introduction

The composition equivalent method is widely used to predict the phase type of steels since last century. The equivalent method is achieved with the aid of a constitution diagram covering austenite, ferrite, martensite and their overlapped phase fields. The chemical contributions of alloying elements in the diagram are evaluated by two composition equivalents, a chromium equivalent (Cr_eq_) and a nickel equivalent (Ni_eq_), which are expressed as the equivalent stability contribution of each alloying element in terms of Cr or Ni, respectively. All alloying elements in steels are henceforth grouped into Ni-like and Cr-like elements, so-called austenitic and ferritic stabilizers, and their contributions to Cr_eq_ and Ni_eq_ are weighted by certain empirical coefficients obtained from mass of experiments. A phase-type prediction can therefore be made by following the phase field in the constitution diagram as specified by the Cr_eq_ and Ni_eq_ coordinates. It is obvious that the accuracy of the constitution diagram and the assorted equivalent equation determine the phase-type predictability of the equivalent method.

**Figure 1:**
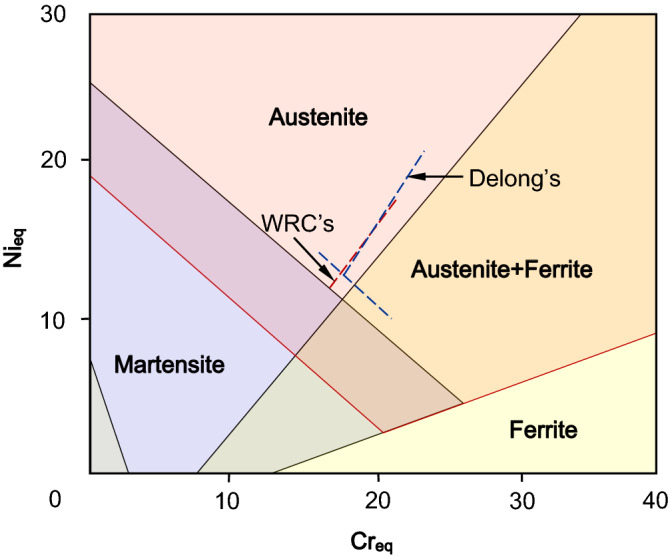
Original Schaeffler constitution diagram^1^, showing modified boundaries of austenite zone and its overlapped zones with ferrite and martensite by Delong^2^ (dashed blue lines) and WRC^8^ (dashed red line).

The most well-known constitution diagram is Schaeffler diagram^1^ developed initially for stainless steel welding. Schaeffler diagram (Fig. 1) contains mainly phase fields of austenite, martensite, ferrite and their overlapped zones, with iso-ferrite lines for the prediction of ferritic percentage. However, only a few alloying elements are counted in Schaeffler’s equivalent equations^1^, Ni_eq_ = Ni + 30C + 0.5Mn, Cr_eq_ = Cr + Mo + 1.5Si + 0.5Nb, where the coefficient represents the relative contribution of each element to austenitic or ferritic stability. With the development of stainless steels, more accurate phase-type predication for complex compositions is demanded. New constitution diagrams and relevant equivalent equations were proposed, generally targeting specific stainless steels with specified range of compositions^2–11^. The equivalent method no longer just predicts welding microstructure but also is widely used to measure the phase stability of any steels as functions of their chemical compositions. For example, it is used to design stainless steels with desired ferrite contents^3^, to predict martensite content with various carbon content^10^, and to guarantee enough austenite stability for fine mechanical properties while pursuing good corrosion resistance^12^.

The improvements to original Schaeffler’s equivalent method were carried out in two aspects: one is the modification to the constitution diagram, in particular the iso-ferrite lines, and the other is the correction to the equivalent equations. Significant modifications to the boundaries of pure austenite zone and its overlapped zones with ferrite and martensite were made by Delong^2^, shown as dashed blue lines in Fig. 1. On this basis, constitution diagrams with further modified boundary of austenite and duplex zones for weld metals were published by Welding Research Council in 1992^8^, shown as the dashed red line in Fig. 1. It is noted that only a small area in the original Schaeffler diagram, around those dashed lines, have been modified. Further modifications^13–16^ has been made to the boundary separating austentite and austenite + martensite zones in the constitution diagarams, though they only apply to specific compositions. In contrast to the few modifications to the constitution diagram, the equivalent equations are extensively addressed^2–11^, as listed in Table 1. The contributions of (N, Co, Cu) and (W, V, Al, Ti, Ta) were respectively added to Ni_eq_ and Cr_eq_ on the basis of original Schaeffler’s equivalent equations. The equivalent coefficients of the same elements may be different. For example, the ferritic stability contribution of Al was claimed to be 2.48 times that of Cr by Hull^3^, but Pickering^5^ gave 5.5 and Tchizhik et al.^9^ suggested 2.8. Another example is that the coefficients for N claimed by different researchers vary from 18 to 30. No new equivalent equations have been put forwards since the twenty-first century^17–25^, signifying the maturity of the equivalent method.

**Table 1:** A summary of coefficient of each element in Ni/Cr equivalents referring to literature^1–11^, and last two lines provide the range of our calculated coefficients and of the corresponding contents.

References	Ni_eq_								Cr_eq_							
Year/researcher	Ni	Co	Mn	N	C	Cu	Cr	Mo	W	Si	V	Nb	Al	Ti	Ta	Co
1949, Schaeffler	1		0.5		30		1	1		1.5		0.5				
1973, Delong	1		0.5	30	30		1	1		1.5		0.5				
1973, Hull	1	0.41	0.11–0.0086^2^	18.4	24.5	0.44	1	1.21	0.72	0.48	2.27	0.14	2.48	2.2	0.21	
1979, Hammar et al	1		0.3	14.2	22	1	1	1.37		1.5		2		3		
1978, Pickering	1	1	0.5	25	20	0.3	1	1.5	0.75	2	5	1.75	5.5	1.5		
1986, Cheng et al	1		0.5		30		1	1	0.75	1.5	1.3	0.5				
1987, Sasmal	1		0.5		30		1	1.76	0.97	1.58	2.02	1.7		2.44	1.22	− 0.177
1988, WRC	1			20	35		1	1				0.7				
1992, WRC	1			20	35	0.25	1	1				0.7				
1998, Tchizhik et al	1	0.5	0.5	30	40	0.3	1	1	0.5	1.5	2.5	1.5	2.8	2		
1998, Beres	1		0.5		10 + 0.2/C		1	1		1.5						
1999, Uggowitzer et al	1	1	0.1–0.01^2^	18	30		1	1.5	1.5	0.48	2.3	1.75	2.5			
Calculated coefficients	1		0.9–3.2	9.2–16.7	9.3–31.8	0.8–1.8	1	1–2.3	0.9–1.4	1.8–3.8	2.4–2.5	2.1	3.5–7.1	4.3–7.9	1.5	0–0.1
Calculated content range, wt. %	0.5–32.5		0.2–14.5	0.01–0.36	0.03–1.08	0.2–4	9–26	0.3–6	1–3	0.1–2.5	0.2–0.4	0.1–1	0.1–1.2	0.2–3	0.1	0.2–20

For the diversification of equivalent coefficients, Raghavan^26^ proposed a reasonable explanation that the equivalent coefficient should vary primarily with the alloying content (i.e., with Ni and other alloying elements) and to a less extent with processing (i.e., with annealing temperature and cooling rate). Because of the composition-dependent coefficient, a reliable prediction is made mainly for steels with similar chemistry, and the selection of proper equivalent equations for new steels becomes difficult. What’s more, the coefficients are derived from experiments under diverse conditions. A deep understanding about the theoretical origin of the equivalent method is therefore required.

Actually the constitution diagram is a special representation of non-equilibrium (i.e., processing dependent) phase diagram in a multi-element system, containing phase fields and their boundaries. The phase-type prediction can then be accessed via the phase field boundary of a specific phase in a certain multi-element phase diagram. Schaeffler diagram is found to approximately agree with isothermal Fe–Cr–Ni phase diagrams covering austenite and ferrite zones^27^. This is why we interpreted the equivalent coefficient of certain alloying element as the slope of the phase field boundary line relative to that of the major alloying element, as exemplified in Mo equivalent for β-Ti alloys^28^. The average slope of the boundary line separating β and β + α phase zones in a Ti-based binary phase diagram is taken as the contribution of the element to the β phase stability. A large slope means a strong stabilizing effect of this element. This slope is then divided by that of Mo to obtain the stability capability of this element relative to that of Mo. In this way, the contributions from each alloying elements are obtained and the new Mo equivalent, termed (Moeq)_Q_, better explains the structural stability of β-Ti alloys and aids the development of novel multi-component β-Ti alloys with low Young’s modulus.

The most important concept in prevailing equivalent method is that the contribution of a certain element is only related to its content (i.e., the slope of boundary line depends on its given composition) but independent of the presence of other alloying elements (i.e., the expansion and shrinkage of phase boundaries caused by any other alloying element are ignored). It is also the fundamental concept in our equivalent method and has been proved by Brandi^27^ through thermodynamic calculation. This concept is justified for classical alloys which are mostly terminal solid solutions; it may not hold for high entropy alloys in which interactions among alloying elements cannot be ignored. This simplification comes from the difficulty in assessing the structural stability in multi-element alloys, even with aid of phase diagram calculation tools. On that basis, the slope of a specified lower boundary of γ phase zone in the Fe-M (M is any alloying element) binary phase diagram, is considered as the actual phase stabilizing efficiency of any alloying element. Different from our previous equivalent based on the average slope of the entire phase boundary, here the slope of selected phase boundary, starting from pure Fe and ending at the given composition, is applied.

This slope of an alloying element, which is determined by its intrinsic characteristic and content, effectively evaluates its phase stabilizing ability in stainless steels. In this way, the effect of alloying content is taken into consideration, which explains the diversified equivalent coefficients and a more accurate prediction of phase stability can be achieved. More importantly, the slope of lower boundary of γ phase zone in the Fe-M binary phase diagram binary phases are regarded as a theoretical origin of equivalent coefficient. And on this basis the coefficient of each alloying element in a steel with given composition can be calculated without distinguishing austenitic or ferritic stabilizer.

This paper firstly defines a γ stabilizing efficiency *k*_M_ of alloying element M in stainless steels, which refers to the γ/γ + α phase boundary slope at a given M content in Fe-M binary diagram. By analyzing as many as 118 standard stainless steels, the equivalent coefficients of the alloying elements are then calculated according to this newly defined γ stabilizing efficiency. Finally, the calculated coefficients are used to show the 118 stainless steels on Schaeffler diagram to further prove the feasibility of γ stabilizing efficiency to evaluate the phase stability of alloying elements.

## Definition of γ stabilizing efficiency

In the conventional equivalent method, the structure of any steel is measured by two parameters, Ni_eq_ and Cr_eq_, which reflect respectively the stabilities of γ and α phases. Using these two parameters, in combination with experimentally obtained phase zones, the constitution diagrams are constructed for the phase-type prediction of steels. Therefore, such constitution diagrams can be regarded as a simplified representation of multi-element phase diagrams, using only two composition inputs. In fact, the high-temperature γ phase stability is the key in determining the overall structural stability of steels. This γ stability is measured by the decomposition boundary of γ phase zone towards α phase zone. Henceforth, the phase-type prediction of steels becomes the description of this boundary in multi-element system, which is highly difficult both by experiments and by phase diagram calculations.

In prevailing equivalent method, the contribution from any element is only individual, independent of the presence of other elements. We here also assume that the γ decomposition boundary in a complex alloy system is determined by weighted contributions of all alloying elements. This is a reasonable assumption because the probability of an alloying element interacting with the base solvent Fe is much higher than the probabilities interacting among alloying elements themselves. That is why the Fe-M binary diagram is used to investigate the phase stabilizing ability of an alloying element M.

**Figure 2:**
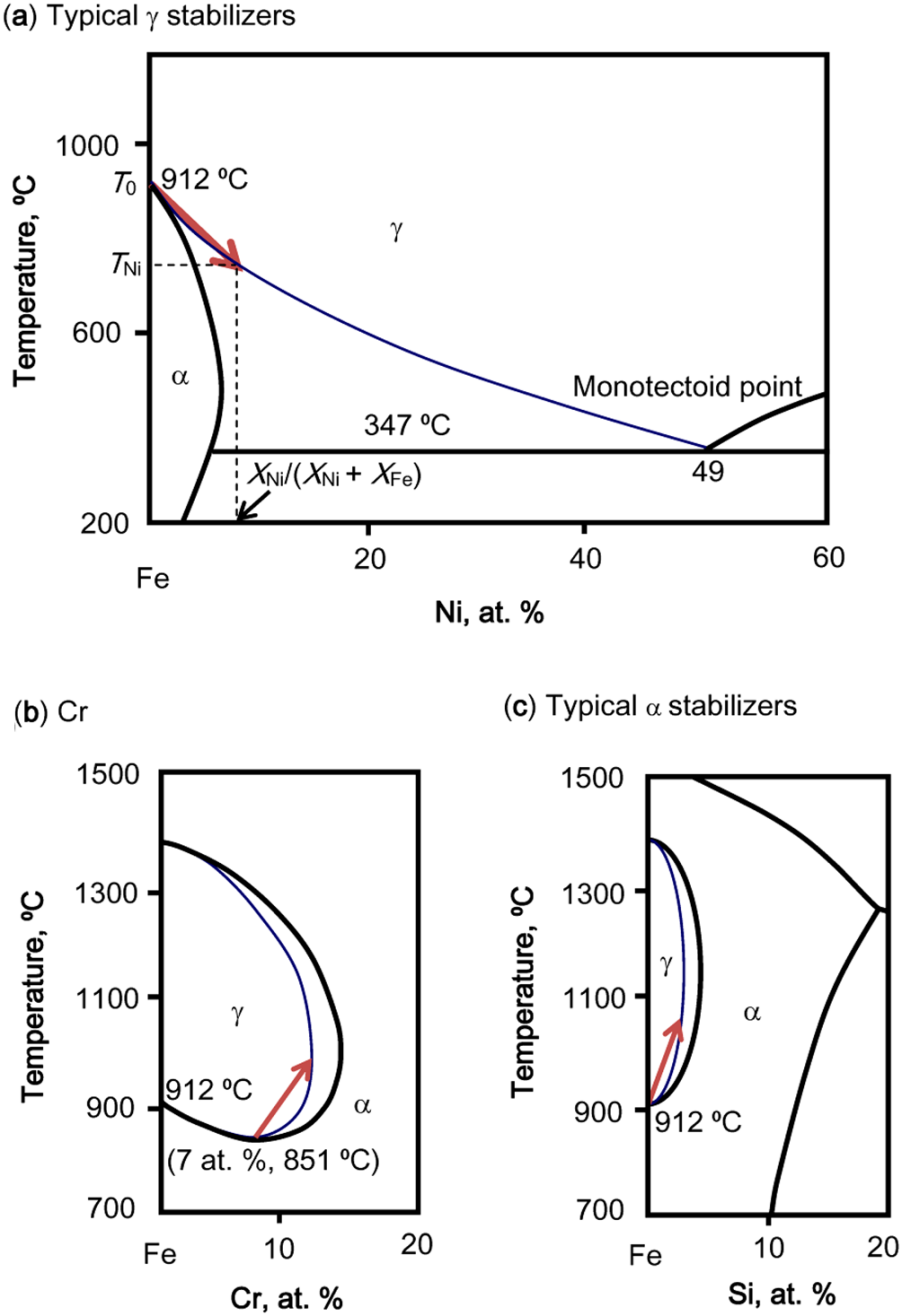
Fe-rich sides of binary phase diagrams of Fe–Ni (**a**), Fe–Cr (**b**), and Fe-Si (**c**), showing the γ decomposition boundaries (blue lines) and the slope of the lower boundary of γ phase zone (red lines).

Unlike the commonly used equivalent coefficient which treats the γ/α stabilizing ability of an alloying element as constant, we here stress that its phase stabilizing ability is influenced by its chemical content. The γ decomposition boundary, or the γ/γ + α phase zone boundary can be obtained in Fe-based binary phase diagram. This is schematically exemplified in Fig. 2a in Fe-rich side of Fe–Ni binary phase diagram. The γ decomposition boundary is generally curved. In Fe–Ni binary system, a certain amount (*X*_Ni_) of alloying element Ni induces a γ decomposition temperature *T*_Ni_, which reflects its γ stabilizing contribution. More importantly, the slope of the lower boundary of γ phase zone, *k*_M_, reflects the γ stabilizing efficiency of M. It should be noticed, for Fe-based alloys, *X*_M_/(*X*_M_ + *X*_Fe_) which reflects the proportion of M in Fe-M binary system, is used to calculate the slope *k*_M_, where *X*_M_ is the percentage of M. A linear approximation (red line) is drawn from the pure Fe point at *T*_0_ = 912 °C to (*X*_Ni_/(*X*_Ni_ + *X*_Fe_), *T*_Ni_) on the lower boundary of γ phase zone. The slope *k*_Ni_, (*T*_Ni_ − *T*_0_)/[*X*_Ni_/(*X*_Ni_ + *X*_Fe_)] measures the average efficiency of Ni addition in stabilizing γ. So that the γ stabilizing efficiency of Ni (i.e. the slope of red line as shown in Fig. 2a) can be expressed as *k*_Ni_ = (*T*_Ni_ − *T*_0_)/[*X*_Ni_/(*X*_Ni_ + *X*_Fe_)], where the temperature difference between initial decomposition temperature of pure Fe (*T*_0_ = 912 °C) and the actually one upon alloying (*T*_Ni_) indicates the expansion effect of the γ zone upon *X*_Ni_/(*X*_Ni_ + *X*_Fe_) alloying. As a result, a high *k*_Ni_ corresponds to a large and negative temperature difference *T*_Ni_ − *T*_0_ and low *X*_Ni_/(*X*_Ni_ + *X*_Fe_). In general, the γ stabilizing efficiency of any alloying element M is expressed as *k*_M_ = (*T*_M_ − *T*_0_)/[*X*_M_/(*X*_M_ + *X*_Fe_)]. A negative slope value *k*_M_ means enhanced γ stability, and a large one indicates a highly efficient γ stabilizing ability of M (for example. Ni is a good γ stabilizer). For instance, a typical 304 stainless steel containing 9.5 wt% Ni (or 8.8 at.% Ni) and its relevant *X*_Ni_/(*X*_Ni_ + *X*_Fe_) = 12.2 wt% or 11.7 at.% expanding the γ zone down to 686 °C, so that the *k*_Ni_ equals to (686 – 912)/12.2 = − 18.5 °C/wt% Ni or (686 – 912)/11.7 = − 19.3 °C/at.% Ni. While in 310S containing a much higher Ni content of 20.5 wt% Ni or 19 at.% Ni, *T*_Ni_ reaches 515 °C and the *k*_Ni_ becomes − 13.9 °C/wt% Ni or − 14.4 °C/at.% Ni. Thus it can be seen that γ stabilizing efficiency for an alloying element is not constant but changes with its content, which provides an explanation of the various equivalent coefficients reported in the literature. It should also be pointed out that, in spite of the changes, the γ stabilizing efficiency of Ni in any cases is quite large and falls within a certain range, say – 16 to − 22 °C/wt% Ni, which justifies why a constant assignment of Ni coefficient can still work in most cases.

On the other hand, elements destabilizing γ shrink the γ phase zones, encircled by ferrite α phase zone, as exemplified by Cr and Si. Cr shows a two-stage behavior. Before 7 at.% Cr, Cr expands the boundary and afterwards shrinks it into a closed loop, as shown in Fig. 2b. The linear approximations of this γ loop of the two-stage boundary line are defined respectively by linking *T*_0_ = 912 °C at the pure Fe origin to the lowest decomposition limit 851 °C at 6.55 wt% Cr (or 7 at.% Cr), and by linking this latter point to 1015 °C at 11.17 wt% Cr (or 11.9 at.% Cr), the decomposition limit in terms of composition. The corresponding γ stabilizing efficiencies are respectively − 9.3 °C/wt% (or − 8.7 °C/at.%) and 35.5 °C/wt% (or 33.5 °C/at.%) Cr, signifying a γ stabilizing and a γ destabilizing process. In stainless steels, the contents of Cr usually surpass 7 at.% Cr to guarantee corrosion resistance so that Cr is generally regarded as a γ destabilizer (or α stabilizer). As a result, the γ stabilizing efficiency of Cr, *k*_Cr_, is evaluated according to the second stage of its lower boundary of γ phase zone, shown as the slope of the red line in Fig. 2b. In stainless steels, with the addition of γ stabilizers, this γ loop is expanded, leading to larger solubility limits of Cr in γ phase. However, the slope of the second stage basically remains unchanged even after the expansion of γ phase zone, so does its γ stabilizing efficiency. In fact, the relative Cr contents, *X*_Cr_/(*X*_Cr_ + *X*_Fe_), of common stainless steels are always larger than 11.9 at.%, so that *k*_Cr_ is regarded as constant, as 33.5 °C/at.% or 35.5 °C/wt%.

Si exemplifies for all the other γ destabilizers, with its continuous shrinking effect on γ phase zone, as shown in Fig. 2c. For 304 stainless steel containing 1 wt% Si (or 1.94 at.% Si), *X*_Si_/(*X*_Si_ + *X*_Fe_) = 1.44 wt% (or 2.83 at.%) and *T*_Si_ = 1046 °C, therefore *k*_Si_ is 93 °C/wt% Si (or 47.4 °C/at.% Si). Si has a maximum γ destabilizing efficiency of 136.2 °C/wt% Si (or 69.9 °C/at.% Si), reaching *T*_Si_ = 1153 °C at the solubility limit of 1.77 wt% (or 3.45 at.%) *X*_Si_/(*X*_Si_ + *X*_Fe_)_,_ beyond which *k*_Si_ stays constant as 136.2 °C/wt% Si. Si therefore has a strong γ destabilizing efficiency, about 3.8 times in wt% (or 2.1 times in at.%) that of Cr when 1.77 wt% or more Si is added.

In brief, the slope in any binary alloy system Fe-M, *k*_M_, is expressed as the γ decomposition temperature difference of an alloyed steel with respect to that of pure Fe (generally 912 °C, except 851 °C for Cr), divided by the proportion of M with respect to Fe + M, *X*_M_/(*X*_M_ + *X*_Fe_), where *X*_M_ and *X*_Fe_ represent respectively the percentage of M and Fe in the steel. There are basically two kinds of alloying elements, austenite stabilizers and destabilizers (ferrite stabilizers), respectively with negative and positive γ stabilizing efficiency *k*_M_.

## Coefficients calculated by γ stabilizing efficiency

In equivalent method, the contribution of each alloying element to the structural stability of a certain phase (i.e. γ/α for steel) is evaluated by an equivalent coefficient multiplied by its content. This indicates that equivalent coefficient actually gives the γ stabilizing efficiency *k*_M_ value of an element relative to that of the major alloying element (i.e. Ni or Cr for stainless steel). Therefore the equivalent coefficient of any alloying element M with a given content can be calculated based on the γ stabilizing efficiency. For any steel with a given composition, the equivalent coefficient of a γ or α stabilizer M equals to the *k*_M_ ratio of M with respect to Ni or Cr. Since *k*_M_ is content-dependent, the thus derived equivalent coefficient should change with the contents of M and of the main components Ni and Cr in steels. However, the *k*_Cr_ stays constant as 35.5 °C/wt% in stainless steels as previously discussed. For stainless steels, the calculated coefficients should only be affected by the contents of M and Ni, rather than Cr, which is consistent with the investigation by Raghavan^26^. This calculation method of equivalent coefficients provides a reasonable theoretical explanation for the variation of equivalent coefficients in different steels.

**Figure 3:**
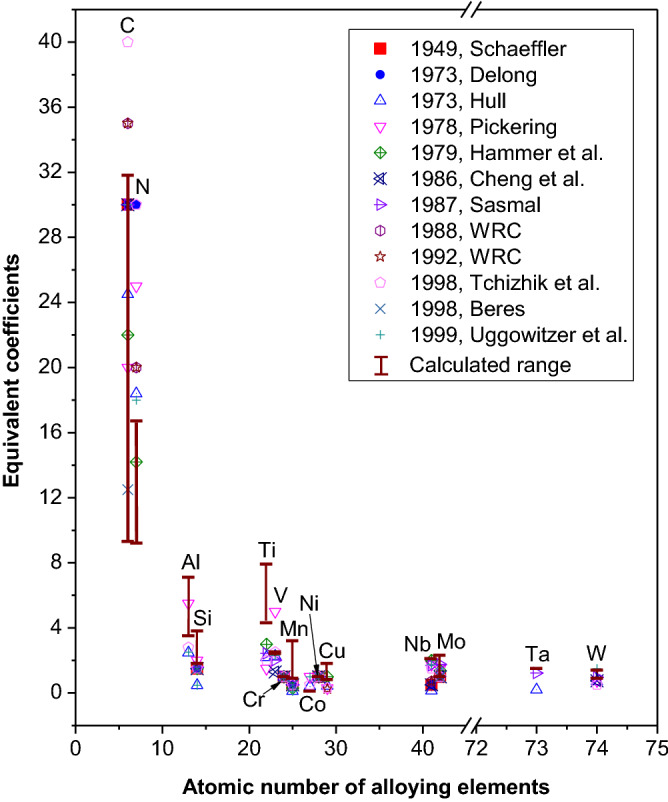
Weight percent equivalent coefficients of the literature^1–11^ (symbols) and of the 118 common stainless steels calculated using γ stabilizing efficiency (solid-line segments).

The equivalent coefficients of alloying elements in 118 common stainless steels (see Supplementary Table S1 online), are calculated using the γ stabilizing efficiency *k*_M_. The range of all calculated coefficients are listed in Table 1 and plotted in Fig. 3, together with the empirical ones^1–11^. It should be noticed that *k*_M_ in wt% is used to calculate the equivalent coefficients in order to compare with the prevailing ones. It is shown that the calculated coefficients agree well with the prevailing ones, which proves that the *k*_M_ is reliable for the evaluation of the stabilizing ability. It is noted specifically that the equivalent coefficient of any element, both prevailing and calculated, covers a certain range. The ranges are especially broad for C and N, which means the equivalent coefficients of C and N are strongly correlated with their contents and with the coefficient of Ni. For instance, the calculated equivalent coefficient of C is 28.1 for 310S and 21.9 for 304 stainless steel, which is consistent with the prevailing equivalent coefficients ranges from 20 to 40^1–11^. Similarly, the prevailing equivalent coefficient of N covers a range of 18 to 30^2–5,8,9,11^ and our calculation show a range from 9.2 to 16.7. The equivalent coefficients of other alloying elements vary in relatively small ranges. Among them, the equivalent coefficient of Al changes more pronouncedly, from 2.5 to 5.5 according to references^3,5,9,11^ and from 3.5 to 7.1 as calculated for the 118 common stainless steels. In general, the range of our calculated composition-dependant coefficients matches well with the prevailing coefficients, as shown in Fig. 3.

**Figure 4:**
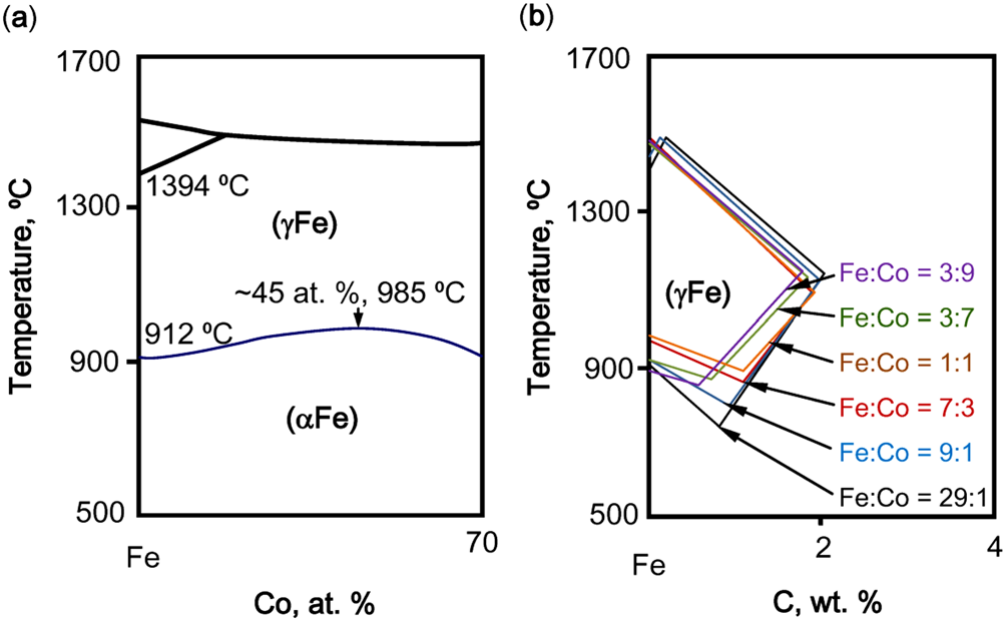
(**a**) Fe-rich side of Fe-Co binary phase diagram, showing the lower boundary of γ phase zone (blue line). (**b**) Fe-C-Co ternary phase diagram at a series of Fe:Co = 29:1, 9:1, 7:3, 1:1, 3:7, 3:9 (wt%), showing the gradual change of γ phase zone^29^.

Among all elements, Co deserves a special attention. In prevailing equivalent equations, Co is regarded as a γ stabilizer, with its coefficients ranging between 0.5 and 1. However, the *k*_Co_ calculated according to Fe-Co binary diagram is a positive and small value, indicating a weak γ destabilizing effect. As shown in Fe-Co binary phase diagram in Fig. 4a, adding Co to pure Fe forms a continuous solid solution, that is to say Co extends infinitely the γ zone, proving its γ stabilizing tendency in terms of composition. On the other hand, from 0 to 45 at.% Co, the lower boundary of γ phase zone slightly rises with the addition of Co, which indicates its weak γ destabilizing effect in terms of temperature. Similarly in Fe-C-Co ternary phase diagram shown in Fig. 4b, the area of γ phase zone slightly shrinks with the amounts of Co increasing, which means that the addition of Co decreases the γ stability of Fe-C-Co ternary alloy system, just as *k*_Co_ indicates. In conclusion, Co is regarded as a weak γ destabilizer and then classified into Cr series.

What is more, in this paper, only the equivalent coefficients of 118 common stainless steels are calculated, for they are widely used and well investigated. The calculation can well be extended to any steels, such as low alloy steels and high-temperature steels. Therefore this new calculating method of the equivalent coefficient, based on the slope of lower boundary of γ phase zone, not only explains the variation of empirical data but also generalizes equivalent coefficients into all kinds of steels.

## Analysis of stainless steels using calculated coefficients

**Figure 5:**
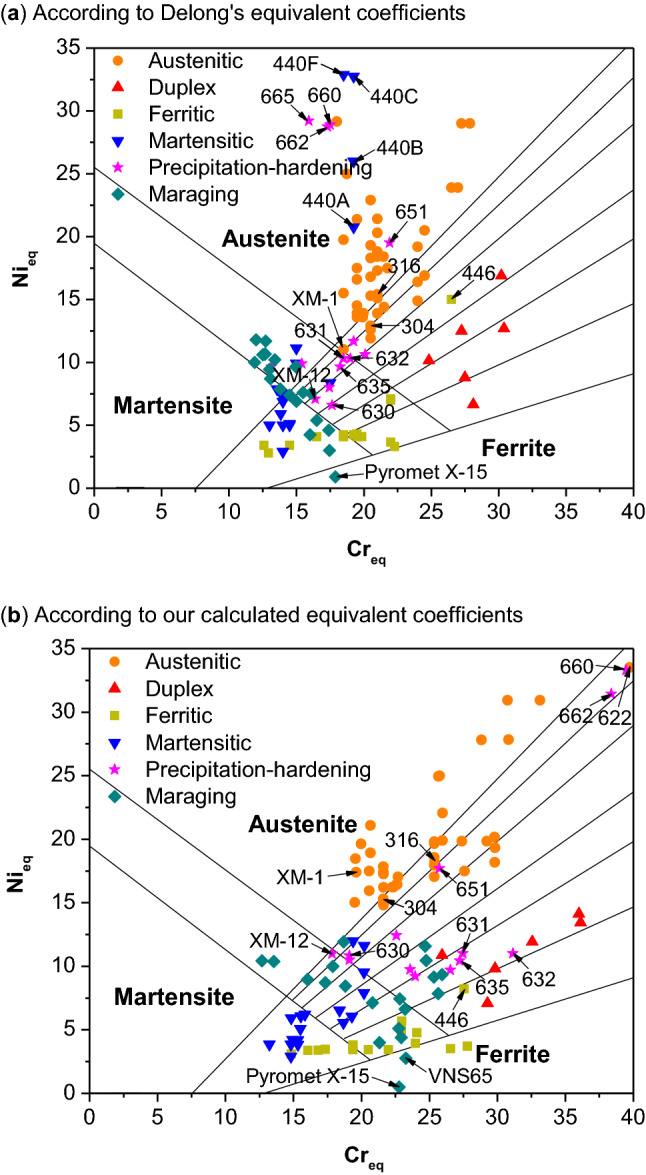
Common stainless steels analyzed by Schaeffler constitution diagram, according to (**a**) Delong’s equivalent coefficients; (**b**) our calculated equivalent coefficients.

For comparison, common stainless steels are analyzed by Delong’s (a) and our calculated equivalent coefficients (b), respectively, and plotted on Schaeffler diagram, as shown in Fig. 5. Here we use Delong’s equation instead of Schaeffler’s as a reference because it involves nitrogen, a strong γ stabilizer, which should not be ignored. As many as 118 compositions, including 100 standard stainless steels^30^ published by American Society for Testing and Materials (ASTM) and 18 maraging stainless steels^31^ are analyzed. The entire list of 118 compositions can be found as Supplementary Table S1 online and the content ranges of alloying elements are listed in Table 1. From a general view, different types of stainless steels distribute similarly on Schaeffler diagram when Delong’s and our coefficients are applied. It proves that our calculated equivalent coefficients are reliable for phase-type prediction of stainless steels.

In Fig. 5a, martensitic 440 series show extremely high Ni_eq_, even higher than most austenitic stainless steels. This is caused by the high content of C (i.e. 0.675–1.075 wt%) plus the high coefficient of C (i.e. equal to a constant 30) suggested by Delong. The equivalent coefficient of C in Delong’s equation seems overestimate the stabilizing effect of C in 440 series stainless steels. However, according to our composition-dependent coefficients, the equivalent coefficient of C is only 9.3 with such high C contents. As a consequence, their extraordinary high Ni_eq_ is significantly reduced, and the location of series 440 falls out of the pure austenite zone as shown in Fig. 5b. The same applies to ferritic 446 with a C content of 0.2 wt%: it is shifted to pure ferrite zone after using the calculated coefficients.

Another advantage is that our calculated equivalent coefficients well separate martensitic and semi-austenitic precipitation-hardening stainless steels, in good matching with Schaeffler diagram. In contrast, the mixed situation occurs when Delong’s coefficients are applied (as shown in Fig. 5a). As shown in Fig. 5b, the martensitic type, XM-12 and 630, are now located near the martensite-ferrite–austenite overlapping zone, while the semi-austenitic ones, 631, 632 and 635, are located in austenite plus ferrite zone.

In conclusion, the γ stability efficiency well classifies common stainless steels and better evaluates their phase stability. However, there is still a big challenge in dealing Co containing steels: this element is considered a good austenite stabilizer, similar to Ni, but in our approach, it destabilizes slightly austenite, on the contrary. For this reason, maraging stainless steels Pyromet X-15 and VNS65, with high Co contents, are located in ferrite region. Further modifications are necessary with regard to the definition of γ stabilizing efficiency of Co: Co indeed slightly shrinks the austenite zone in terms of temperature but nevertheless expands the zone to infinity in terms of Co content.

## Conclusions

In order to understand the variation of equivalent coefficients of stainless steels, a γ stabilizing efficiency of an alloying element M is defined as *k*_M_ = (*T*_M_ − *T*_0_)/[*X*_M_/(*X*_M_ + *X*_Fe_)]. It is derived from the slope of the lower boundary of γ phase zone in a Fe-M binary phase diagram. A negative *k*_M_ indicates a γ stabilizer (i.e. Ni, Mn, N, C, and Cu), while positive one suggests a α stabilizer (i.e. Cr, Mo, W, Si, V, Al, Ti, Ta, Nb, Co). For common stainless steels, a constant *k*_Cr_ (i.e., the slope at this Cr limit) is assumed, while *k*_Ni_ varies with its content, for instance, − 18.5 °C/wt% Ni for 304 and − 13.9 °C/wt% Ni for 310S. The prevailing equivalent coefficients are well interpreted by *k*_M_/*k*_Ni_ for γ stabilizers and *k*_M_/*k*_Cr_ for α stabilizers, after analyzing 118 common stainless steels. Because of the composition-dependent γ stabilizing efficiency *k*_M_, the thus-derived coefficients also vary with compositions, which explain the variety of prevailing equivalent coefficients. Furthermore, this new parameter *k*_M_ evaluates the absolute phase stabilizing efficiency of any alloying element; no matter it is a γ stabilizer or a α stabilizer.

## Supplementary Information

Supplementary Information.

## Data Availability

The datasets generated during and/or analyzed during the current study are available from the corresponding author on reasonable request.
