# Supplementary material for: Composition equivalents of stainless steels understood via gamma stabilizing efficiency
Source: Sci Rep. 2021 Mar 8;11:5423. doi: 10.1038/s41598-021-84917-z (PMC7940414; doi:10.1038/s41598-021-84917-z)
Supplement: Supplementary file 1 — Supplementary Information. [file 41598_2021_84917_MOESM1_ESM.docx]

SUPPLEMENTARY INFORMATION

**Composition equivalents of stainless steels understood via gamma stabilizing efficiency**

Shuqi Zhang, Qing Wang, Rui Yang, Chuang Dong

**Supplementary Table S1.** Chemical compositions (in wt. %) of 118 stainless steels analyzed by γ stabilizing efficiency, including 100 standard stainless steels^1^ published by American Society for Testing and Materials (ASTM) and 18 maraging stainless steels^2^.

| **Grade** | **C** | **Si** | **Mn** | **S** | **P** | **Cr** | **Mo** | **Ni** | **Cu** | **N** | **Others** |
| --- | --- | --- | --- | --- | --- | --- | --- | --- | --- | --- | --- |
| **201** | 0.15 | 1.00 | 5.5-7.5 | 0.03 | 0.06 | 16.0-18.0 |  | 3.5-5.5 |  | 0.25 |  |
| **202** | 0.15 | 1.00 | 7.5-10.0 | 0.03 | 0.06 | 17.0-19.0 |  | 4.0-6.0 |  | 0.25 |  |
| **205** | 0.12-0.25 | 1.00 | 14.0-15.0 | 0.03 | 0.06 | 16.5-18.0 |  | 1.0-1.75 |  | 0.34-0.40 |  |
| **301** | 0.15 | 1.00 | 2.00 | 0.03 | 0.045 | 16.0-18.0 |  | 6.0-8.0 |  | 0.1 |  |
| **302** | 0.15 | 1.00 | 2.00 | 0.03 | 0.045 | 17.0-19.0 |  | 8.0-10.0 |  | 0.1 |  |
| **302B** | 0.15 | 2.0-3.0 | 2.00 | 0.03 | 0.045 | 17.0-19.0 |  | 8.0-10.0 |  | 0.1 |  |
| **303** | 0.15 | 1.00 | 2.00 | 0.15 | 0.2 | 17.0-19.0 |  | 8.0-10.0 |  |  |  |
| **303Se** | 0.15 | 1.00 | 2.00 | 0.06 | 0.2 | 17.0-19.0 |  | 8.0-10.0 |  |  | Se 0.15min |
| **304** | 0.08 | 1.00 | 2.00 | 0.03 | 0.045 | 18.0-20.0 |  | 8.0-11.0 |  |  |  |
| **304H** | 0.04-0.10 | 1.00 | 2.00 | 0.03 | 0.045 | 18.0-20.0 |  | 8.0-11.0 |  |  |  |
| **304L** | 0.03 | 1.00 | 2.00 | 0.03 | 0.045 | 18.0-20.0 |  | 8.0-12.0 |  |  |  |
| **304N** | 0.08 | 1.00 | 2.00 | 0.03 | 0.045 | 18.0-20.0 |  | 8.0-11.0 |  | 0.10-0.16 |  |
| **XM-21** | 0.08 | 1.00 | 2.00 | 0.03 | 0.045 | 18.0-20.0 |  | 8.0-10.0 |  | 0.16-0.30 |  |
| **304LN** | 0.03 | 1.00 | 2.00 | 0.03 | 0.045 | 18.0-20.0 |  | 8.0-11.0 |  | 0.10-0.16 |  |
| **304LHN** | 0.03 | 1.00 | 2.00 | 0.03 | 0.045 | 18.0-20.0 |  | 8.0-11.0 |  | 0.16-0.30 |  |
| **305** | 0.12 | 1.00 | 2.00 | 0.03 | 0.045 | 17.0-19.0 |  | 11.0-13.0 |  |  |  |
| **308** | 0.08 | 1.00 | 2.00 | 0.03 | 0.045 | 19.0-21.0 |  | 10.0-12.0 |  |  |  |
| **309** | 0.2 | 1.00 | 2.00 | 0.03 | 0.045 | 22.0-24.0 |  | 12.0-15.0 |  |  |  |
| **309S** | 0.08 | 1.00 | 2.00 | 0.03 | 0.045 | 22.0-24.0 |  | 12.0-15.0 |  |  |  |
| **310** | 0.25 | 1.50 | 2.00 | 0.03 | 0.045 | 24.0-26.0 |  | 19.0-22.0 |  |  |  |
| **310S** | 0.08 | 1.00 | 2.00 | 0.03 | 0.045 | 24.0-26.0 |  | 19.0-22.0 |  |  |  |
| **310Nb** | 0.08 | 1.00 | 2.00 | 0.03 | 0.045 | 24.0-26.0 |  | 19.0-22.0 |  |  | Nb 10C-1.10 |
| **314** | 0.25 | 1.5-3.0 | 2.00 | 0.03 | 0.045 | 23.0-26.0 |  | 19.0-22.0 |  |  |  |
| **316** | 0.08 | 1.00 | 2.00 | 0.03 | 0.045 | 16.0-18.0 | 2.0-3.0 | 10.0-14.0 |  |  |  |
| **316H** | 0.07 | 1.00 | 2.00 | 0.03 | 0.045 | 16.0-18.0 | 2.0-3.0 | 10.0-14.0 |  |  |  |
| **316L** | 0.03 | 1.00 | 2.00 | 0.03 | 0.045 | 16.0-18.0 | 2.0-3.0 | 10.0-14.0 |  |  |  |
| **316Ti** | 0.08 | 1.00 | 2.00 | 0.03 | 0.045 | 16.0-18.0 | 2.0-3.0 | 10.0-14.0 |  | 0.1 | Ti 5(C+N)-0.70 |
| **316Nb** | 0.08 | 1.00 | 2.00 | 0.03 | 0.045 | 16.0-18.0 | 2.0-3.0 | 10.0-14.0 |  | 0.1 | Nb 10C-1.10 |
| **316N** | 0.08 | 1.00 | 2.00 | 0.03 | 0.045 | 16.0-18.0 | 2.0-3.0 | 10.0-13.0 |  | 0.10-0.16 |  |
| **316LN** | 0.03 | 1.00 | 2.00 | 0.03 | 0.045 | 16.0-18.0 | 2.0-3.0 | 10.0-13.0 |  | 0.10-0.16 |  |
| **316LHN** | 0.03 | 1.00 | 2.00 | 0.03 | 0.045 | 16.0-18.0 | 2.0-3.0 | 10.0-13.0 |  | 0.16-0.30 |  |
| **317** | 0.08 | 1.00 | 2.00 | 0.03 | 0.045 | 18.0-20.0 | 3.0-4.0 | 11.0-15.0 |  |  |  |
| **317LMN** | 0.03 | 1.00 | 2.00 | 0.03 | 0.045 | 18.0-20.0 | 3.0-4.0 | 11.0-15.0 |  |  |  |
| **317LN** | 0.03 | 1.00 | 2.00 | 0.03 | 0.045 | 18.0-20.0 | 3.0-4.0 | 11.0-14.0 |  | 0.1-0.22 |  |
| **321** | 0.08 | 1.00 | 2.00 | 0.03 | 0.045 | 17.0-19.0 |  | 9.0-12.0 |  |  | Ti 5(C+N)-0.70 |
| **321H** | 0.04-0.10 | 1.00 | 2.00 | 0.03 | 0.045 | 17.0-19.0 |  | 9.0-12.0 |  |  | Ti 4(C+N)-0.70 |
| **334** | 0.08 | 1.00 | 1.00 | 0.015 | 0.03 | 18.0-20.0 |  | 19.0-21.0 |  |  | Ti 0.51-0.60,Al 0.15-0.60 |
| **347** | 0.08 | 1.00 | 2.00 | 0.03 | 0.045 | 17.0-19.0 |  | 9.0-12.0 |  |  | Nb 10C-1.10 |
| **347H** | 0.04-0.10 | 1.00 | 2.00 | 0.03 | 0.045 | 17.0-19.0 |  | 9.0-12.0 |  |  | Nb 8C-1.10 |
| **348** | 0.08 | 1.00 | 2.00 | 0.03 | 0.045 | 17.0-19.0 |  | 9.0-12.0 |  |  | Nb+Ta=10C-1.10, Co 0.2 |
| **XM-1** | 0.08 | 1.00 | 5.0-6.5 | 0.18-0.35 | 0.045 | 16.0-18.0 |  | 5.0-6.5 |  |  | Cu 1.75-2.25 |
| **XM-15** | 0.08 | 1.5-2.5 | 2.00 | 0.03 | 0.03 | 17.0-19.0 |  | 17.5-18.5 |  |  |  |
| **622** | 0.08 | 1.00 | 1.50 | 0.03 | 0.04 | 12.0-15.0 | 2.5-3.5 | 24.0-28.0 | 0.5 |  | Cu 0.5,Ti1.55-2.0,B 0.03-0.10,Al 0.35 |
| **800** | 0.1 | 1.00 | 1.50 | 0.015 | 0.045 | 19.0-23.0 |  | 30.0-35.0 | 0.75 |  | Cu 0.75,Al 0.15-0.6,Ti 0.15-0.6 |
| **XM-26** | 0.06 | 1.00 | 1.00 | 0.03 | 0.045 | 25.0-27.0 |  | 6.0-7.0 |  |  | Ti 0.25 |
| **329** | 0.08 | 0.75 | 1.00 | 0.03 | 0.04 | 23.0-28.0 | 1.0-2.0 | 2.5-5.0 |  |  |  |
| **2205** | 0.03 | 1.00 | 2.00 | 0.02 | 0.03 | 22.0-23.0 | 3.0-3.5 | 4.5-6.5 |  | 0.14-0.20 |  |
| **2304** | 0.03 | 1.00 | 2.50 | 0.03 | 0.04 | 21.5-24.5 | 0.05-0.60 | 3.0-5.5 | 0.05-0.60 | 0.05-0.20 | Cu 0.05-0.60, |
| **2507** | 0.03 | 0.80 | 1.20 | 0.02 | 0.035 | 24.0-26.0 | 3.0-5.0 | 6.0-8.0 | 0.5 | 0.24-0.32 | Cu 0.5 |
| **255** | 0.04 | 1.00 | 1.50 | 0.03 | 0.04 | 24.0-27.0 | 2.9-3.9 | 4.5-6.5 | 1.5-2.5 | 0.10-0.25 | Cu 1.5-2.5 |
| **XM-34** | 0.08 | 1.00 | 2.50 | 0.15min | 0.04 | 17.5-19.5 | 1.5-2.5 |  |  |  |  |
| **405** | 0.08 | 1.00 | 1.00 | 0.03 | 0.04 | 11.5-14.5 |  | 0.5 |  |  | Al 0.1-0.3 |
| **409** | 0.08 | 1.00 | 1.00 | 0.03 | 0.045 | 10.5-11.7 |  | 0.5 |  |  | Ti 6C-0.75 |
| **409Nb** | 0.06 | 1.00 | 1.00 | 0.04 | 0.045 | 10.5-11.7 |  | 0.5 |  |  | Nb 10C-0.75 |
| **429** | 0.12 | 1.00 | 1.00 | 0.03 | 0.04 | 14.0-16.0 |  |  |  |  |  |
| **430** | 0.12 | 1.00 | 1.00 | 0.03 | 0.04 | 16.0-18.0 |  |  |  |  |  |
| **430F** | 0.12 | 1.00 | 1.25 | 0.15 min | 0.06 | 16.0-18.0 |  |  |  |  |  |
| **430FSe** | 0.12 | 1.00 | 1.25 | 0.06 | 0.06 | 16.0-18.0 |  |  |  |  | Se 0.15min |
| **430Ti** | 0.1 | 1.00 | 1.00 | 0.03 | 0.04 | 16.0-19.5 |  | 0.75 |  |  | Ti 5C-0.75 |
| **434** | 0.12 | 1.00 | 1.00 | 0.03 | 0.04 | 16.0-18.0 | 0.75-0.15 |  |  |  |  |
| **436** | 0.12 | 1.00 | 1.00 | 0.03 | 0.04 | 16.0-18.0 | 0.75-0.15 |  |  |  | Nb 5C-0.8 |
| **439** | 0.07 | 1.00 | 1.00 | 0.03 | 0.04 | 17.0-19.0 |  | 0.5 |  | 0.04 | Al 0.15,Ti 0.20+4(C+N)-1.10 |
| **442** | 0.2 | 1.00 | 1.00 | 0.04 | 0.04 | 18.0-23.0 |  | 0.6 |  |  |  |
| **443** | 0.2 | 1.00 | 1.00 | 0.03 | 0.04 | 18.0-23.0 |  | 0.5 | 0.9-1.25 |  | Cu 0.9-1.25 |
| **444** | 0.025 | 1.00 | 1.00 | 0.03 | 0.04 | 17.5-19.0 | 1.75-2.5 | 1 |  | 0.035 | Ti+Nb=0.20+4(C+N)-0.80 |
| **446** | 0.2 | 1.00 | 1.50 | 0.03 | 0.04 | 23.0-27.0 |  | 0.75 |  | 0.25 |  |
| **PH13-8Mo** | 0.05 |  |  |  |  | 12.2-13.2 | 2.0-2.5 | 7.5-8.5 |  | 0.005 | Al 1.2 |
| **Custom450** | 0.035 |  |  |  |  | 14.9 | 0.8 | 6.5 | 1.5 |  | Cu 1.5,Nb 0.75 |
| **Custom455** | 0.05 |  |  |  |  | 11.0-12.5 |  | 7.5-9.5 | 2.25 |  | Cu 2.25,Ti 0.8,Nb 0.3 |
| **Custom465** | 0.02 |  |  |  |  | 11.0-12.5 | 0.75-1.2 | 11.0-11.2 |  |  | Ti 1.5 |
| **Pyromet X-23** | 0.02 |  |  |  |  | 9.5-10.5 | 5.0-6.0 | 6.5-7.5 |  |  | Co 9.5-11 |
| **Pyromet X-15** | 0.03 |  |  |  |  | 15 | 2.9 | 0 |  |  | Co 20 |
| **Custom475** | 0.03 |  |  |  |  | 9 | 6 | 6 |  |  | Co 14 |
| **D70** | 0.03 |  |  |  |  | 11.5-12.5 | 4.0-5.0 | 4.0-5.0 |  |  | Co 12.0-14.0 |
| **03Kh11N10M2T** | 0.03 |  |  |  |  | 10.45 | 2.24 | 9.77 |  |  | Ti 1.3 |
| **VNS25** | 0.03 |  |  |  |  | 11.5-12.5 | 0.5-0.8 | 9.0-10.5 |  |  |  |
| **VNS65** | 0.02 |  |  |  |  | 13.4-13.6 | 3.9-4.0 | 2.2-2.6 |  |  | Co 16.1-16.3 |
| **VNS59** | 0.03 |  |  |  |  | 9.5-10.5 | 3.2-3.6 | 8.8-9.8 |  |  | Co 6.5-7.5 |
| **Almar362** | 0.03 |  |  |  |  | 14.5 |  | 6.5 |  |  | Ti 0.8 |
| **AM367** | 0.025 |  |  |  |  | 14 | 2 | 3.5 |  |  | Ti 0.4,Co 15.5 |
| **000KHl4N4Kl4M3T** | 0.02 |  |  |  |  | 14.5 | 2.9 | 4 |  |  | Ti 0.26,Co 14.6 |
| **03Kh11N10M2T2** | 0.03 |  |  |  |  | 10.5 | 2 | 9.7 |  |  |  |
| **03Kh12N8K5M3TYu** | 0.0028 |  |  |  |  | 11.5 | 2.3 | 7.8 |  |  | Ti 0.26,Al 0.33,Co 5.3 |
| **0Khl2N8MTYu** | 0.03 |  |  |  |  | 12.2 | 0.88 | 7.8 |  |  | Al 0.8 |
| **11Cr9NiMoTi** | 0.015 |  |  |  |  | 11 | 2 | 9 |  |  | Ti 1.5,B 0.005 |
| **FerriumS53** | 0.21 |  |  |  |  | 10 | 2 | 5.5 |  |  | Co 14,W 1,V 0.3 |
| **403** | 0.15 | 0.50 | 1.00 | 0.03 | 0.04 | 11.5-13.0 |  |  |  |  |  |
| **410S** | 0.08 | 1.00 | 1.00 | 0.03 | 0.04 | 11.5-13.5 |  |  |  |  |  |
| **410** | 0.15 | 1.00 | 1.00 | 0.03 | 0.04 | 11.5-13.5 |  |  |  |  |  |
| **410Nb** | 0.18 | 1.00 | 1.00 | 0.03 | 0.04 | 11.5-13.0 |  |  |  |  | Nb 0.05-0.3 |
| **414** | 0.15 | 1.00 | 1.00 | 0.03 | 0.04 | 11.5-13.5 |  | 1.25-2.50 |  |  |  |
| **416** | 0.15 | 1.00 | 1.25 | 0.15min | 0.06 | 12.0-14.0 |  |  |  |  |  |
| **416Se** | 0.15 | 1.00 | 1.25 | 0.06 | 0.06 | 12.0-14.0 |  |  |  |  | Se 0.15min |
| **615** | 0.15-0.2 | 0.50 | 0.50 | 0.03 | 0.04 | 12.0-14.0 | 0.5 | 1.8-2.2 |  |  | W 2.5-3.5 |
| **420** | 0.15min | 1.00 | 1.00 | 0.03 | 0.04 | 12.0-14.0 |  |  |  |  |  |
| **420F** | 0.3-0.4 | 1.00 | 1.25 | 0.15min | 0.06 | 12.0-14.0 | 0.5 |  |  |  |  |
| **616** | 0.2-0.25 | 0.50 | 0.5-1.0 | 0.025 | 0.025 | 11.0-12.5 | 0.9-1.25 | 0.5-1.0 |  |  | W 0.9-1.25,V 0.2-0.3 |
| **619** | 0.27-0.32 | 0.50 | 0.95-1.35 | 0.025 | 0.025 | 11.0-12.0 | 2.5-3.0 | 0.5 |  |  | V 0.2-0.3 |
| **431** | 0.2 | 1.00 | 1.00 | 0.03 | 0.04 | 15.0-17.0 |  | 1.25-2.50 |  |  |  |
| **440A** | 0.6-0.75 | 1.00 | 1.00 | 0.03 | 0.04 | 16.0-18.0 | 0.75 |  |  |  |  |
| **440B** | 0.75-0.95 | 1.00 | 1.00 | 0.03 | 0.04 | 16.0-18.0 | 0.75 |  |  |  |  |
| **440C** | 0.95-1.20 | 1.00 | 1.00 | 0.03 | 0.04 | 16.0-18.0 | 0.75 |  |  |  |  |
| **440F** | 0.95-1.20 | 1.00 | 1.25 | 0.15min | 0.06 | 16.0-18.0 |  |  |  |  |  |
| **XM-32** | 0.08-0.15 | 0.35 | 0.5-0.9 | 0.025 | 0.025 | 11.0-12.5 | 1.5-2.0 | 2.0-3.0 |  | 0.01-0.05 | V 0.25-0.40, |
| **XM-13** | 0.05 | 0.10 | 0.20 | 0.008 | 0.01 | 12.3-13.2 | 2.0-3.0 | 7.5-8.5 |  | 0.01 | Al 0.9-1.35 |
| **XM-12** | 0.07 | 1.00 | 1.00 | 0.03 | 0.04 | 14.0-15.5 |  | 3.5-5.5 | 2.5-4.5 |  | Cu 2.5-4.5,Nb 0.15-0.45 |
| **630** | 0.07 | 1.00 | 1.00 | 0.03 | 0.04 | 15.0-17.0 |  | 3.0-5.0 | 3.0-5.0 |  | Cu 3.0-5.0,Nb 0.15-0.45 |
| **631** | 0.09 | 1.00 | 1.00 | 0.03 | 0.04 | 16.0-18.0 |  | 6.5-7.7 |  |  | Al 0.75-1.50 |
| **632** | 0.09 | 1.00 | 1.00 | 0.03 | 0.04 | 14.0-16.0 | 2.0-3.0 | 6.5-7.7 |  |  | Al 0.75-1.50 |
| **633** | 0.07-0.11 | 0.50 | 0.5-1.25 | 0.03 | 0.04 | 16.0-17.0 | 2.5-3.2 | 4.0-5.0 |  | 0.07-0.13 |  |
| **634** | 0.10-0.15 | 0.50 | 0.50-1.25 | 0.03 | 0.04 | 15.0-16.0 | 2.5-3.2 | 4.0-5.0 |  | 0.07-0.13 | Nb 0.10-0.50 |
| **635** | 0.08 | 1.00 | 1.00 | 0.03 | 0.04 | 16.0-17.5 |  | 6.0-7.5 |  |  | Ti 0.4-1.2,Al 0.4 |
| **XM-25** | 0.05 | 1.00 | 1.00 | 0.03 | 0.04 | 14.0-16.0 | 0.5-1.0 | 5.0-7.0 | 1.25-1.75 |  | Cu 1.25-1.75,Nb 8Cmin |
| **XM-16** | 0.03 | 0.50 | 0.50 | 0.03 | 0.04 | 11.0-12.5 | 0.5 | 7.5-9.5 | 1.5-2.5 |  | Nb 0.1-0.5,Ti 0.8-1.4,Cu 1.5-2.5 |
| **651** | 0.28-0.35 | 0.3-0.8 | 0.75-1.50 | 0.03 | 0.04 | 18.0-21.0 | 1.0-1.75 | 8.0-11.0 | 0.5 |  | Cu 0.5, Ti 0.10-0.35,Nb 0.25-0.60,W 1.00-1.75 |
| **662** | 0.08 | 0.4-1.0 | 0.4-1.0 | 0.03 | 0.04 | 12.0-15.0 | 2.0-3.5 | 24.0-28.0 | 0.5 |  | Cu 0.5,Ti 1.80-2.10,B 0.001-0.010,Al 0.35 |
| **660** | 0.08 | 1.00 | 2.00 | 0.03 | 0.04 | 13.5-16.0 | 1.00-1.50 | 24.0-27.0 |  |  | V 0.10-0.50,Ti 1.90-2.35,B 0.001-0.010,Al 0.35 |
| **665** | 0.08 | 0.10-0.80 | 1.25-2.00 | 0.03 | 0.04 | 12.0-15.0 | 1.25-2.25 | 24.0-28.0 | 0.25 |  | Cu 0.25,Ti 2.7-3.2,B 0.01-0.07,Al 0.25 |

Note: Except clearly marked as a minimum or composition range, the composition stands for maximum.

Reference

1 ASTM A959-00a, Standard Guide for Specifying Harmonized Standard Grade Compositions for Wrought Stainless Steels, ASTM International, West Conshohocken, PA, 2001, [www.astm.org](https://www.astm.org/)

2 Yang, Z. Y., Liu, Z. B., Liang, J. X., Sun, Y. Q. & Li, W. H. Development of maraging stainless steel. *Trans. Mater. Heat Treat.* **29**, 3-9 (2008).
